# Microbial diversity of plant pathogens and insect endosymbionts in *Reptalus artemisiae*

**DOI:** 10.1186/s12866-026-04915-x

**Published:** 2026-03-13

**Authors:** Bojan Duduk, Ivana Galic, Nikola Stanojević, Nada Stankovic, Emil Rekanović

**Affiliations:** 1 Institute of Pesticides and Environmental Protection, Banatska 31B, 11080 Belgrade, Serbia; 2 Institute of Molecular Genetics and Genetic Engineering, University of Belgrade, Vojvode Stepe 444a, 11042 Belgrade, Serbia

**Keywords:** *Reptalus quinquecostatus*, Stolbur phytoplasma, Genome, Vector, Plant disease, Pathogen, Metagenomics

## Abstract

**Background:**

Phloem-sap-feeding planthopper *Reptalus artemisiae* is an emerging vector of rubbery taproot disease (RTD) and syndrome basses richesses (SBR) in sugar beet, diseases associated with '*Candidatus* Phytoplasma solani' and '*Candidatus* Arsenophonus phytopathogenicus', respectively. Despite studies on related cixiids, the microbiome of *R. artemisiae* remains uncharacterized. Using a PCR-free metagenomic long-read shotgun sequencing approach, this study investigates the bacterial diversity associated with *R. artemisiae*, and provides genomic insight into two plant pathogens '*Ca.* P. solani' and '*Ca.* A. phytopathogenicus'.

**Results:**

Taxonomic assignment revealed six prokaryotic taxa in *R. artemisiae*: two plant pathogens ('*Ca.* P. solani' and '*Ca.* A. phytopathogenicus') and four insect endosymbionts – three primary endosymbionts ('*Candidatus* Vidania', '*Candidatus* Purcelliella', and '*Candidatus* Karelsulcia') and a secondary endosymbiont (*Wolbachia*). Community profiles showed a consistent presence of all four endosymbionts across five evaluated *R. artemisiae* individuals. Phylogenetic analyses of 16S rRNA gene sequences of primary endosymbionts confirmed strong congruence with the cytochrome oxidase subunit I phylogeny of the insect host, indicative of long coevolution and vertical transmission. In contrast, plant pathogen presence in *R. artemisiae* varied, with '*Ca*. P. solani' and '*Ca*. A. phytopathogenicus' each detected in three individuals. Genome assembly yielded a complete 774 kb circular chromosome for '*Ca.* P. solani' with streamlined metabolism featuring limited biosynthetic pathways, but a full arsenal of genes related to host–pathogen interactions and pathogenicity typical for this biotrophs. The draft genome of '*Ca.* A. phytopathogenicus' comprising 18 scaffolds totalling 3.11 Mb and two plasmids shows a self-sufficient metabolism with several missing metabolic modules and presence of genomic islands, virulence factors, and a dynamic mobilome indicating a bacterium in transition that is reorganizing its genetic material, possibly in response to host interactions.

**Conclusion:**

These findings represent the first in-depth characterization of *R. artemisiae* microbiome, highlighting a stable endosymbiont consortium and variable pathogen presence that emphasize ecological complexity in vector-pathogen-endosymbiont interactions. The assembled genomes enhance the understanding of microbial ecology, pathogen adaptation and transmission, offering resources for comparative genomics and potential applications in disease management strategies.

**Supplementary Information:**

The online version contains supplementary material available at 10.1186/s12866-026-04915-x.

## Introduction

Phloem-limited plant pathogenic prokaryotes are generally non-cultivable, fastidious, and strictly vector-borne. These pathogens are transmitted from plant to plant by phloem-feeding insect vectors belonging to the suborders Auchenorrhyncha and Sternorrhyncha of the order Hemiptera [26, 29]. Responsible for some of the most devastating plant diseases worldwide, including two economically important diseases that hinder sugar beet production in Europe, phloem-limited plant pathogenic prokaryotes pose a significant threat to agriculture. The predominant insect-borne disease affecting sugar beet in the Pannonian Plain, rubbery taproot disease (RTD), is associated with '*Candidatus* Phytoplasma solani', a wall-less bacterium belonging to the class Mollicutes [18, 19, 22]. Another phloem-limited plant pathogen, '*Candidatus* Arsenophonus phytopathogenicus' associated with syndrome basses richesses (SBR) of sugar beet in France, Germany, and Switzerland, has recently been detected in the Pannonian Plain. Although both pathogens are reportedly transmitted by the cixiid planthopper *Pentastiridius leporinus* Linnaeus in France and Germany, the main vector of the phytoplasma on sugar beet in the Pannonian Plain is another cixiid planthopper, *Reptalus artemisiae* (Becker, 1865), which was also recently reported in Austria as capable of transmitting '*Ca*. A. phytopathogenicus' to sugar beet [4, 12, 38, 40, 62]. The cixiid planthopper *R. artemisiae* (Becker, 1865) was historically reported under the name *Reptalus quinquecostatus* (Dufour, 1833) until a taxonomic revision by Webb et al. [82] rejected the latter and, consequently, *R. artemisiae* was proposed as the valid name for this taxon. Although the species’ nomenclature remains contested pending further taxonomic clarification, we have adopted the name *R. artemisiae* (Becker, 1865) for the species previously referred to as *R. quinquecostatus* (Dufour, 1833) sensu Holzinger et al. [32], in accordance with recent nomenclature revisions [23, 72, 82].

Phloem-limited plant pathogenic bacteria are transmitted in a persistent, propagative manner, colonizing and replicating within their insect vectors to integrate into the complex endosymbiont community of the host’s microbiota. These pathogens not only modulate the physiology and behaviour of their vectors but also engage in intricate interactions with co-occurring microbial populations within the insect. Such interactions, which may be antagonistic or synergistic, can profoundly influence the insect host’s fitness and pathogen transmission efficiency [1, 26, 29, 63]. According to their dependence on the insect host, bacterial endosymbionts are classified as either primary (obligate) or secondary (facultative) [57]. Primary endosymbionts are vertically (matrilineally) transmitted and provide the insect host with nutrients lacking in its phloem-sap-feeding diet, typically essential amino acids and vitamins [21, 52] that are indispensable for host growth and reproduction [14]. The distinction between plant pathogens and secondary insect endosymbionts is often blurred. Notably, species of the genus *Arsenophonus* are considered both plant pathogens and secondary endosymbionts of insects [56, 57]. The colonization of a single insect individual by multiple bacterial symbionts/plant pathogens has been reported for the vector cixiids *P. leporinus* and *Hyalesthes obsoletus* [11, 20, 29, 49, 78]. In addition to vectored '*Ca*. P. solani', three primary endosymbionts (β-proteobacterium '*Ca*. Vidania fulgoridae', γ-proteobacterium '*Ca*. Purcelliella pentastirinorum', and flavobacterium '*Ca*. Karelsulcia muelleri') and a secondary endosymbiont (α-proteobacterium *Wolbachia*) have been reported in *H. obsoletus*. In *P. leporinus*, alongside vectored '*Ca.* P. solani' and '*Ca.* A. phytopathogenicus'*,* two facultative symbionts, *Rickettsia* and *Wolbachia*, as well as primary symbionts of the genera '*Ca.* Purcelliella', '*Ca.* Karelsulcia', and '*Ca.* Vidania' have been reported [78]. The aforementioned endosymbionts (*Purcelliella*, *Vidania*, *Karelsulcia* and *Wolbachia*) were also found in other, non-vector cixiids, i.e., *Hyalesthes luteipes*, *Pentastira rorida,* and *Cixius nervosus* [49].

The availability of data on microbial communities of other cixiids, including both vectors and non-vectors of plant-pathogenic bacteria, is limited; however, such information is lacking for the phloem-sap-feeding planthopper *R. artemisiae*, the vector of RTD and SBR pathogens. This study employs a PCR-free metagenomic approach with singleplex long-read shotgun sequencing to investigate the bacterial diversity associated with the insect and characterize its microbiome by identifying insect endosymbionts and plant pathogens. Multiplex barcoding in a single flow cell was used in separate sequencing runs to generate long-read sequences from four *R. artemisiae* specimens, enabling simultaneous characterization of their microbial communities. In addition, the current study obtained genomic insights into two plant pathogens, '*Ca.* A. phytopathogenicus' and '*Ca.* P. solani', enabling a better understanding of their metabolic capacities, key virulence factors, genome plasticity, and pathways that may underlie their establishment in plant and insect hosts.

## Material and methods

### Insect material, DNA extraction, identification, and microbial detection

Cixiids were collected from sugar beet fields and adjacent vegetation in the South Banat region ( northern Serbia). The cixiids were collected during June 2024 within a ~ 3 km radius of an area (N 44^o^56′18’’; E 20^o^43′35’’) with known RTD occurrences, where a '*Ca.* P. solani'-infected *R. artemisiae* population had been recorded in previous years [18, 39]. The cixiids were collected using an entomological sweep net and a mouth aspirator, and stored in 96% ethanol until subjected to morphological and molecular identification and assessment of the presence of phytoplasmas and '*Ca.* A. phytopathogenicus'.

Initial identification of males as *R. artemisiae* and females at the genus level (*Reptalus* sp.), performed with a stereo microscope was based on external morphological features: the size of the body, five distinct longitudinal carinae on the mesonotum, and two transverse keels on the head and, in the case of male morphology, of the anal tube and presence of a left-orientated process [32]. The final identification of insects as *R. artemisiae* was performed molecularly and based on differences in the length of internal transcribed spacer 2 (ITS2) amplicons [7]. Total nucleic acid extraction from individual insects was done following the CTAB protocol [27]. The isolated nucleic acid was resuspended in TE buffer and stored at −20 °C until further analysis. Primer pair ITS2fw/ITS2rv [17] was used for PCR amplification in a 25 μL final reaction volume containing 0.5 μL of template DNA (isolated as described below), 1 × PCR Master Mix (Thermo Scientific, Vilnius, Lithuania) and 0.4 µM of each primer under previously described thermal conditions [7]. Six microlitres of PCR products were separated in a 1% agarose gel, stained with ethidium bromide, and visualized with a UV transilluminator.

### Library preparation and sequencing

Five *R. artemisiae* specimens (two males and three females) were randomly selected for metagenomic analyses. Extracted metagenomic DNA of *R. artemisiae* specimen 135/24 was enriched for longer fragments (> 600 bp) using the Select-a-Size DNA Clean & Concentrator MagBead Kit (Zymo Research, Irvine, CA, USA) according to the manufacturer’s instructions, while the metagenomic DNA of other *R. artemisiae* specimens was used without fragment size enrichment. Metagenomic DNA (200 ng per sample) was used to prepare sequencing libraries with ONT Rapid Barcoding Kit V14 (SQK-RBK114; Oxford Nanopore Technologies, Oxford, UK) according to the manufacturer’s instructions. The single barcoded library for *R. artemisiae* 135/24 was loaded on a FLO-MIN114 flow cell (R10.4.1 Kit 14 sequencing chemistry) and sequenced. Metagenomic DNA of *R. artemisiae* specimen 93/24 was equimolarly pooled as one of the three barcoded libraries and sequenced on a separate flow cell in the first multiplex run, while three barcoded libraries (metagenomic DNA of *R. artemisiae* specimens 92/24, 67/24, and 69/24) were equimolarly pooled for the second multiplex run on the same cell. Sequencing was carried out for 48 h in separate flow cells for *R. artemisiae* 135/24 and *R. artemisiae* 93/24, while the second run of the latter flow cell (applied previously for *R. artemisiae* 93/24) with three pooled barcoded samples was carried out for 71 h. Sequencing was performed on MinION Mk1B with MinKNOW v. 23.11.4 (ONT) on a desktop PC, with super-accurate basecalling, de-multiplexing, and Minimal Q score 10 settings. The raw sequencing data obtained from *R. artemisiae* 135/24 have been deposited in NCBI’s Sequence Read Archive (SRA) and are accessible through BioProject ID PRJNA1215974. Raw metagenomic reads of *R. artemisiae* 135/24 were additionally quality-filtered using Filtlong v. 0.2.1 (https://github.com/rrwick/Filtlong) with a mean quality score of 12, and by omitting the worst 10% of reads based on quality. Adapter trimming was subsequently performed using Porechop v.0.2.4 (https://github.com/rrwick/Porechop).

### Taxonomic profiling

The following analyses were carried out individually for metagenomic DNA of each *R. artemisiae* specimen. Using the DIAMOND high-throughput aligner v. 2.1.9.163 [13] and applying the ‘more sensitive’ parameter, reads (> 500 nt) were compared against a custom-made subset of protein databases containing all proteins assigned to plant pathogens and insect endosymbionts previously associated with cixiids [29]: class Mollicutes, genera '*Candidatus* Arsenophonus', '*Ca.* Vidania', '*Ca.* Purcelliella', '*Ca.* Karelsulcia', and *Wolbachia*, as well as the plant *Catharanthus roseus* and Fulgoridae family. DIAMOND outputs were parsed by using the Metagenome Analyzer (MEGAN) v. 6.25.10 [34] for taxonomic binning with default parameters enabling the assignment of reads. The confirmation of assignments obtained with DIAMOND, as well as assignment of the non-assigned reads, was performed using NCBI BLASTn [35].

For identification of bacterial endosymbionts, complete 16S rRNA gene sequences were extracted from metagenomic reads obtained for *R. artemisiae* 135/24, and aligned with reference sequences and sequences of closely related species (selected by applying the MegaBLAST algorithm, NCBI), using ClustalX, under MEGA version X [43, 71]. The obtained complete 16S rRNA gene sequences were deposited in the NCBI GenBank under accession numbers PX502305- PX502307. Evolutionary history based on 16S rRNA gene sequences obtained in this study and reference strains of the closest species was inferred separately for each insect endosymbiont using the maximum-likelihood (ML) method (MEGA X) and best-fit substitution model. Initial trees for the heuristic search were obtained automatically by applying the Neighbor-Join and BioNJ algorithms. The closest species were used as an outgroup taxon to root the trees. To estimate the statistical significance of the inferred clades, 1,000 bootstraps were performed. To confirm the insect identification, the complete cytochrome oxidase subunit I (*COI*) gene sequence was extracted from reads obtained for *R. artemisiae* 135/24 and aligned with reference sequences, including records with specimen vouchers when available (e.g., *Reptalus quinquecostatus* GQ397852; specimen voucher L7150), and other available sequences of cixiid species as described above. The obtained *COI* sequence of *R. artemisiae* 135/24 was deposited in the NCBI GenBank under accession number PX501388. Evolutionary history based on the *COI* sequences obtained in this study and those of available cixiid species was inferred as described above. The closest species were used as an outgroup taxon to root the trees. The statistical significance of the inferred clades was estimated as described above.

### Genome assembly and functional annotation

To reconstruct the chromosomes of '*Ca*. A. phytopathogenicus' strain 135/24 and '*Ca*. P. solani' strain 135/24 from *R. artemisiae* 135/24 the selections of trimmed reads after barcode removal were assembled to contigs, with six iterations of polishing, using Flye assembler v. 2.9.5 [37]. For '*Ca*. P. solani' two contigs (with identical overlap < 1000 bp) produced in Flye were merged into a single circular scaffold using Genious Prime 2025.2.2. All statistics from the Flye report files are based on contigs of size ≥ 500 bp (except "# contigs (≥ 0 bp)" and "Total length (≥ 0 bp)", which include all contigs). The assemblies generated by Flye were polished using Racon v. 1.54.0 (https://github.com/lbcb-sci/racon) and medaka v. 2.0.1 (https://github.com/nanoporetech/medaka). The chromosomes were annotated using Prokka v. 1.14.6 [65]. The completeness of the assembled genomes was assessed using Busco v. 5.8.0 [67] and CheckM2 [16]. The draft genome sequence of '*Ca.* A. phytopathogenicus' 135/24 and the complete genome sequence of '*Ca.* P. solani' 135/24 from *R. artemisiae* 135/24 have been deposited in the NCBI GenBank under the BioSample accession numbers SAMN51795872 and SAMN51795599, respectively. Functional annotation of the assembled genomes was performed using BlastKOALA [36], which assigned KEGG Orthology (KO) identifiers to input sequences. The PHASTER web server was used to predict prophage regions in the two genomes [2]. Genomic islands (GI) in *Arsenophonus* were predicted and visualised by Islandviewer4 [6] using two GI prediction methods – SIGI-HMM [79], which measures codon usage to identify possible GIs, and IslandPath-DIMOB [33], which visualises common characteristics of GIs such as abnormal sequence composition or the presence of genes that functionally relate to mobile elements. The '*Ca.* A. phytopathogenicus' 135/24 sequences were aligned against the *A. nasoniae* FIN genome, which is the most closely related species with a publicly available complete genome in the IslandViewer tool.

## Results

### Insect selection and identification

**Fig. 1:**
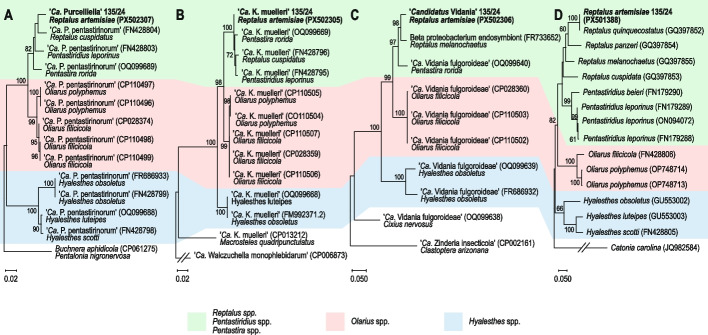
Phylogenetic analyses of 16S rRNA gene sequences of *R. artemisiae* 135/24-associated primary endosymbionts: *Purcelliella* (**A**), *Karelsulcia* (**B**), *Vidania* (**C**), and closely related strains along cytochrome oxidase subunit I (*COI*) gene sequences of *R. artemisiae* 135/24 and closely related cixiid species (**D**). *Cixiid *COI* sequence referred in GenBank as belonging to *R. quinquecostatus*, according to the old nomenclature

Based on external morphological characteristics, two male insect specimens were identified as *R. artemisiae*, while three female specimens were tentatively classified as *Reptalus* sp. Molecular analyses confirmed the identification of all individuals as *R. artemisiae*, based on the specific amplicon size of the ITS2 region, as described previously [7]. Additionally, the cytochrome oxidase subunit I (*COI*) gene sequence extracted from *R. artemisiae* 135/24 was aligned with publicly available sequences, showing the highest similarity (97.4%) to *R. artemisiae*, and thereby corroborating the species-level identification. An ML phylogenetic analysis based on *COI* sequences, including those of the closest related taxa, further supported the taxonomic assignment and was subsequently used for coevolution congruence (co-phylogenetic) analyses with primary endosymbionts (Fig. 1).

### Metagenomic sequencing

To minimize bias in the representation of microbial taxa, non-amplicon long-read shotgun sequencing of *R. artemisiae* specimens metagenomic DNA was performed using the ONT MinION. The overall recovered clean reads differed in each assay—singleplex run, multiplex in the first run, and multiplex in the second run. The singleplex sequencing generated 1,316,420 reads for *R. artemisiae* 135/24 metagenomic DNA in the first run, which corresponded to a total of 2.93 Gbp after base calling. After trimming the adapters and barcodes, the reads had an average length of 3,626 bp. In the first multiplex run on the separate MinION flow cell for metagenomic DNA of *R. artemisiae* 93/24, a total of 383,100 reads (0.46 Gbp) were generated after base-calling, which could be assigned to a barcode with certainty, of which 93,050 reads (0.16 Gb) were assigned to the barcode of metagenomic DNA of *R. artemisiae* 93/24. In the second multiplex, first run on the same flow cell, a total of 66,370 reads (86.59 Mb) were generated after base-calling, which could be confidently assigned to a barcode. For the three pooled barcoded libraries, the distribution of reads across the barcodes was relatively even, ranging from 8.8 to 28.6 Mbp. Sample-level read statistics are shown in Table 1.

**Table 1:** Bacterial distribution across *R. artemisiae* specimens

*R. artemisiae* specimen					Bacteria			
		Reads Total bp	Plant pathogens		Insect endosymbionts			
	Plant host		'Ca. P. solani'	*'Ca.* A. phytopathogenicus'	*Purcelliella*	*Karelsulcia*	*Wolbachia*	*Vidania*
**135/24** ♀	*Prunus spinosa*	678825 2.5 Gb	4646 18.9 Mb	16545 67.7 Mb	3012 12.3 Mb	1577 3.3 Mb	3410 19.7 Mb	1753 4.7 Mb
93/24♀	*Beta vulgaris*	96850 156 Mb	263 550 kb	0	113 199 kb	102 293 kb	465 1.4 Mb	154 166 kb
92/24♀	*Beta vulgaris*	13470 28.6 Mb	82 189 kb	513 987 kb	68 100 kb	30 34 kb	168 432 kb	56 71 kb
67/24♂	*Beta vulgaris*	5945 10 Mb	0	199 255 kb	76 148 kb	27 29 kb	20 64 kb	28 50 kb
69/24♂	*Beta vulgaris*	5995 8.8 Mb	0	0	47 72 kb	6 7 kb	31 90 kb	16 31 kb

*R. artemisiae* 135/24, used for 16S rDNA identification of bacterial strains, is shown in bold

### Microbial community composition

The microbial community of five *R. artemisiae* specimens was analysed through taxonomic assignment of long reads generated by ONT MinION. Taxonomic affiliations were determined using the local DIAMOND aligner against a custom database. Bacterial identification was corroborated by analyzing 16S rRNA gene sequences extracted from *R. artemisiae* 135/24. Further taxonomic assignment revealed six prokaryotic taxa: two plant pathogens ('*Ca*. P. solani' and '*Ca.* A. phytopathogenicus') and four insect endosymbionts (*Vidania*, *Purcelliella*, *Karelsulcia*, and *Wolbachia*). The community profiles across different *R. artemisiae* individuals showed no variability concerning insect endosymbionts, as all four insect endosymbionts were detected in each specimen. In contrast, the occurrence of plant pathogens varied among individual insects, as '*Ca*. P. solani' and '*Ca*. A. phytopathogenicus' were each detected in three specimens of which two had mixed infection.

### Endosymbionts of *R. artemisiae*

Sequences of four insect endosymbionts were detected in all evaluated specimens of *R. artemisiae*. The number of reads assigned to one of the four endosymbionts ranged from as few as six (of 5,995) for *Karelsulcia* to 3,410 (of 678,825) for *Wolba*chia. Sequences of 16S rRNA genes of each endosymbiont were successfully extracted from *R. artemisiae* 135/24 for phylogenetic analysis, whereas only partial or no 16S rRNA gene sequences could be retrieved from the other specimens.

The presence of *Purcelliella* was confirmed in all five analyzed specimens of *R. artemisiae*. Number of reads assigned to *Purcelliella* ranged from 0.36% (37 of 11,015) to 0.44% (3,012 of 678,825) across the individuals. The sequence of the 16S rRNA gene of *Purcelliella* strain 135/24 from *R. artemisiae* 135/24 was aligned with the closest available '*Ca.* P. pentastirinorum' strains, showing the highest similarity (97.4%) to the strain from the closest available host species *R. cuspidatus*. A 16S rDNA-based ML phylogeny confirmed its identification and congruence between the endosymbiont and the insect host species (Fig. 1).

*Karelsulcia* was detected in all five analyzed specimens of *R. artemisiae*. The number of reads assigned to *Karelsulcia* ranged from 0.1% (six of 5,995) to 0.23% (1,577 of 678,825) across the specimens. The alignment of 16S rRNA gene sequences of *Karelsulcia* strain from *R. artemisiae* 135/24 with those of the closest available *Karelsulcia* strains revealed that the former is most similar (> 99%) to strains from available host species most closely related to *R. artemisiae*, i.e. the cixiids *R. cuspidatus* and *Pentastira rorida*. It confirmed identification of *Karelsulcia* strain from *R. artemisiae* 135/24 as '*Ca*. Karelsulcia muelleri' strain 135/24. An ML phylogeny constructed from 16S rRNA gene sequences of '*Ca*. Karelsulcia muelleri' 135/24 aligned with the closest available *Karelsulcia* strains, result in several well-supported branches that correlated with the insect host species. Notably, aside from the well-supported cluster of cixiid *Karelsulcia* strains, two distinct subclusters were formed for strains from the genera *Hyalesthes* and *Oliarus*, wherease strains from the genera *Pentastiridius*, *Pentastira,* and *Reptalus* formed a separate subcluster (Fig. 1).

The number of reads assigned to *Vidania* ranged from 0.06% (seven out of 11,015) to 0.26% (1,753 out of 678,825) across the specimens. The 16S rRNA gene sequence of *Vidania* showed the highest similarity (96.5%) to an unpublished corresponding sequence of a proteobacterium endosymbiont of *Reptalus melanochaetus*. An ML phylogeny constructed from 16S rRNA gene sequences of the *Vidania* strain from *R. artemisiae* 135/24, aligned with the closest available *Vidania* strains, resulted in well-supported clustering that follows the phylogeny of the insect hosts. Aside from a well-supported cluster of strains from the Pentastirini tribe, three additional well-supported clusters were formed—one for *Hyalesthes obsoletus*, one for *Oliarus* sp., and one comprising endosymbionts of genera *Reptalus* and *Pentastira*.

*Wolba*chia was detected in all specimens of *R. artemisiae*, with assigned reads ranging from 0.34% (20 out of 5,945) to 0.5% (3,410 out of 678,825). The obtained 16S rRNA gene sequence of *Wolbachia* from *R. artemisiae* 135/24 showed the highest similarity (96.52%) to those of other *Wolbachia* species (with publicly available full 16S rRNA gene sequences) from *Cixius nervosus*.

Therefore, the phylogenetic analysis of 16S rRNA genes of *R. artemisiae* endosymbionts (*Karelsulcia*, *Purcelliella*, and *Vidania*) confirmed strong congruence between the phylogenies of primary insect endosymbionts and those of the *COI* gene of insect hosts.

### Genome assemblies of plant pathogens

The assembly data of the '*Ca*. P. solani' and '*Ca.* A. phytopathogenicus' genome sequences obtained from *R. artemisiae* 135/24 are shown in Table 2.

**Table 2:** Summary of sequencing and genome assembly statistics for '*Ca*. P. solani' and '*Ca.* A. phytopathogenicus' obtained from *R. artemisiae* 135/24

		'*Ca.* P. solani'	'*Ca*. A. phytopathogenicus'
Raw reads	Total reads before filtering	1316420 (2.93 Gb)	
	Total reads after filtering (nt)	678825 (2.46 Gb)	
	Assigned reads	4646 (18.89 Mb)	16545 (67.73 Mb)
Assembly	Number of contigs	1	20
	Number of circular contigs	1	2
	Extrachromosomal elements (plasmids)	0	2
	Total length (bp)	774234	3110796
	Size of the largest contig	774234	1970366
	Mean coverage	24.4	21
	N50 (bp)	774234	1970366
	N90 (bp)	774234	41261
	L50	1	1
	L90	1	7
	GC%	28.34	37.96
Annotation (Prokka)	Number of Coding Sequences	704	3968
	Number of rRNAs (5S, 16S, 23S)	6 (2, 2, 2)	21 (7, 7, 7)
	Number of tRNAs	32	61
	Number of tmRNAs	1	1
BUSCO analysis (genes)	Total	151	366
	Complete and single-copy	141	339
	Complete and duplicate	1	0
	Fragmented	1	15
	Missing	8	12
NCBI GenBank	BioSample	SAMN51795599	SAMN51795872
Accession Number	BioProject	PRJNA1215974	

### '*Ca*. P. solani' 135/24

Phytoplasmas were detected in three of five analyzed insects. The phytoplasma in *R. artemisiae* 135/24 was identified as '*Ca*. P. solani' by taxonomic assignment of the obtained long reads.

Reads assigned to phytoplasmas ranged from 0.61% (82 of 13,470) to 0.35% (2408 of 678,825) across the specimens. Two rRNA operons, containing identical 16S rRNA gene sequences, were retrieved in *R. artemisiae* 135/24, while in the other specimens, no 16S rRNA gene sequence could be retrieved. The obtained 16S rRNA gene sequence was identical to that of '*Ca*. P. solani' ribosomal group 16SrXII-A reference strain STOL from Serbia, as well as strain c1 from Italy [15]. Analyses of an epidemiologically informative marker, *tuf* gene sequence, classified '*Ca*. P. solani' 135/24 as tuf-D [19].

### Genome assembly and comparative genomic analysis of '*Ca*. P. solani' strain 135/24

**Fig. 2:**
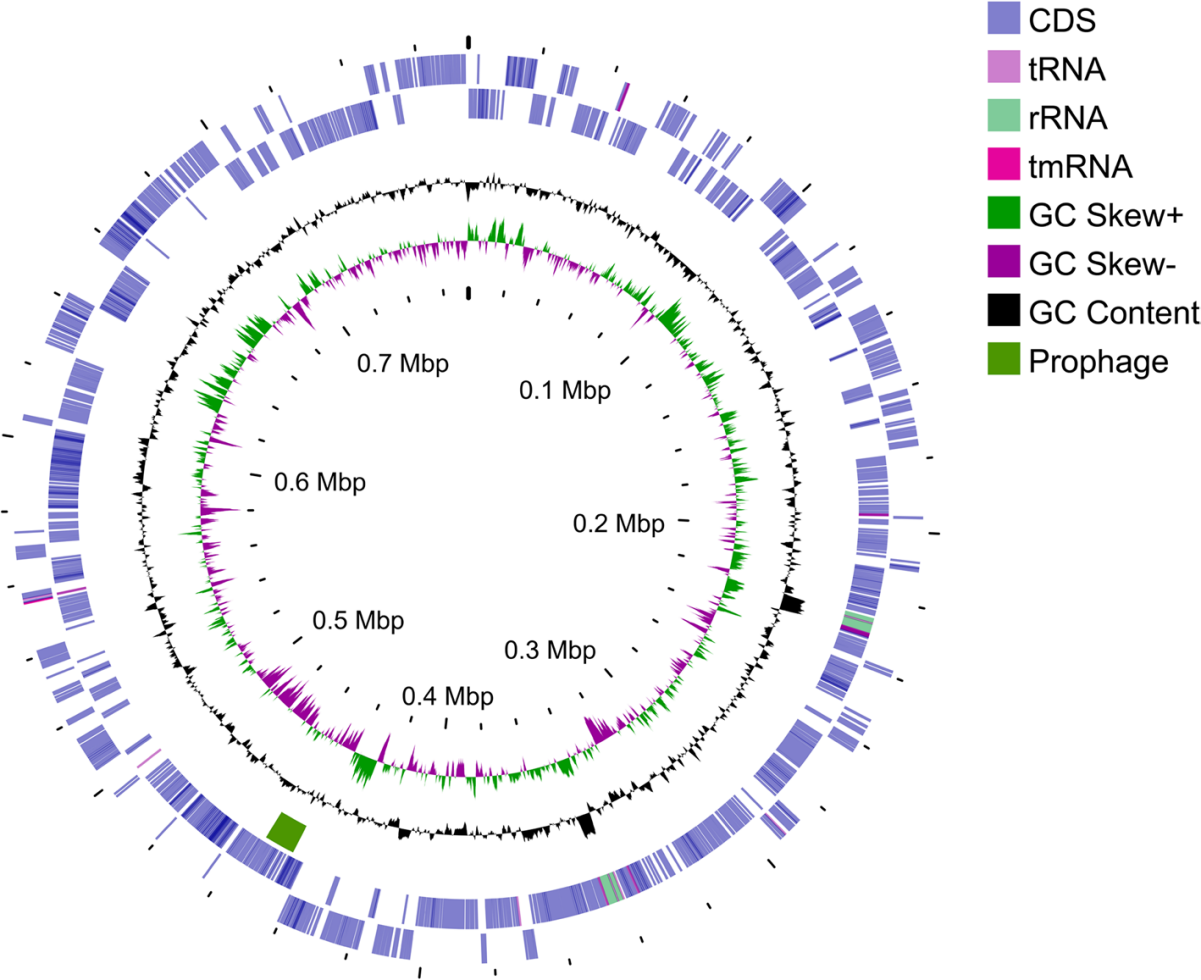
Circular genome map of '*Ca*. P. solani' 135/24 from *R. artemisiae* 135/24

De novo assembly of reads assigned to Mollicutes from the metagenome of *R. artemisiae* 135/24 with six iterations of polishing in Flye produced two contigs, which were merged into a single scaffold with an identical overlap of 246 bp and then circularized in Genious. No extrachromosomal elements were detected. The final circular chromosome of '*Ca*. P. solani' from *R. artemisiae* 135/24 had a total length of 774,238 bp, an average G + C content of 28.34%, and average sequencing depth of 24.4 × (Table 2). The two high GC-content regions located around the 230 and 350 kb positions correspond to the two rRNA operons (Fig. 2). Assessment of the obtained genome completeness using BUSCO showed that among 151 single-copy orthologue genes conserved among Mollicutes (BUSCO database), 142 are present in the obtained genome of '*Ca*. P. solani' 135/24. This indicates high-quality assembly, as reflected by the 94.1% completeness based on the BUSCO analysis. CheckM2 resulted in an even higher level of genome completeness of 99.42%. The assembled chromosome of '*Ca*. P. solani' 135/24 encodes 704 coding sequences, six rRNA genes (two of each 16S rRNA, 23S rRNA, and 5S rRNA, organized into two rRNA operons), 32 tRNA genes and one tmRNA gene (Table 2), representing a set of RNA-coding genes previously reported in phytoplasmas [15, 73].

From outside to inside, the rings represent: (1, 2) predicted protein-coding, rRNA, tRNA, and tmRNA genes on the forward and reverse strands respectively; (3) prophage region determined by PHASTER; (4) GC content; (5) GC skew, with regions above the average shown in green and below average in purple; (6) genome coordinates (kb).

When compared to publicly available genomes of '*Ca*. P. solani', including the reference genome (Acc. no. CP155828), to establish average nucleotide identity, the genome of '*Ca*. P. solani' 135/24 showed close relatedness to strains classified as 16SrXII-A and a more distant relationship with the reference strain GOE 16SrXII-P (Table 3). The main features of '*Ca*. P. solani' 135/24 genome fall within or close to the range of previously published genomes.

**Table 3:** Comparative genomic features and average nucleotide identity (ANI) of publicly available '*Ca*. P. solani' complete genomes and '*Ca*. P. solani' 135/24

	'*Ca*. P. solani' strains					
	**135/24**	c1	c4	c5	o3	GOE
Acc. Number	CM148571	CP103788	CP103787	CP103786	CP103785	CP155828
Ribosomal subgroup	16SrXII-A	16SrXII-A	16SrXII-A	16SrXII-A	16SrXII-A	16SrXII-P
ANI with 135/24 (%)	100	99.08	99.07	99.01	98.47	82.88
Length (bp)	774234	751320	751188	824084	973640	704525
GC (%)	28.3	28.4	28.4	28.1	28.6	26.2
Number of CDS	704	719	724	807	1000	663
Coding density (coding/total bps)	0.773	0.778	0.776	0.787	0.700	0.784
Gene density (genes/kb)	0.910	0.957	0.964	0.979	1.027	0.941
CheckM2 completeness (%)	99.42	99.43	99.42	99.5	99.65	98.65

**Fig. 3:**
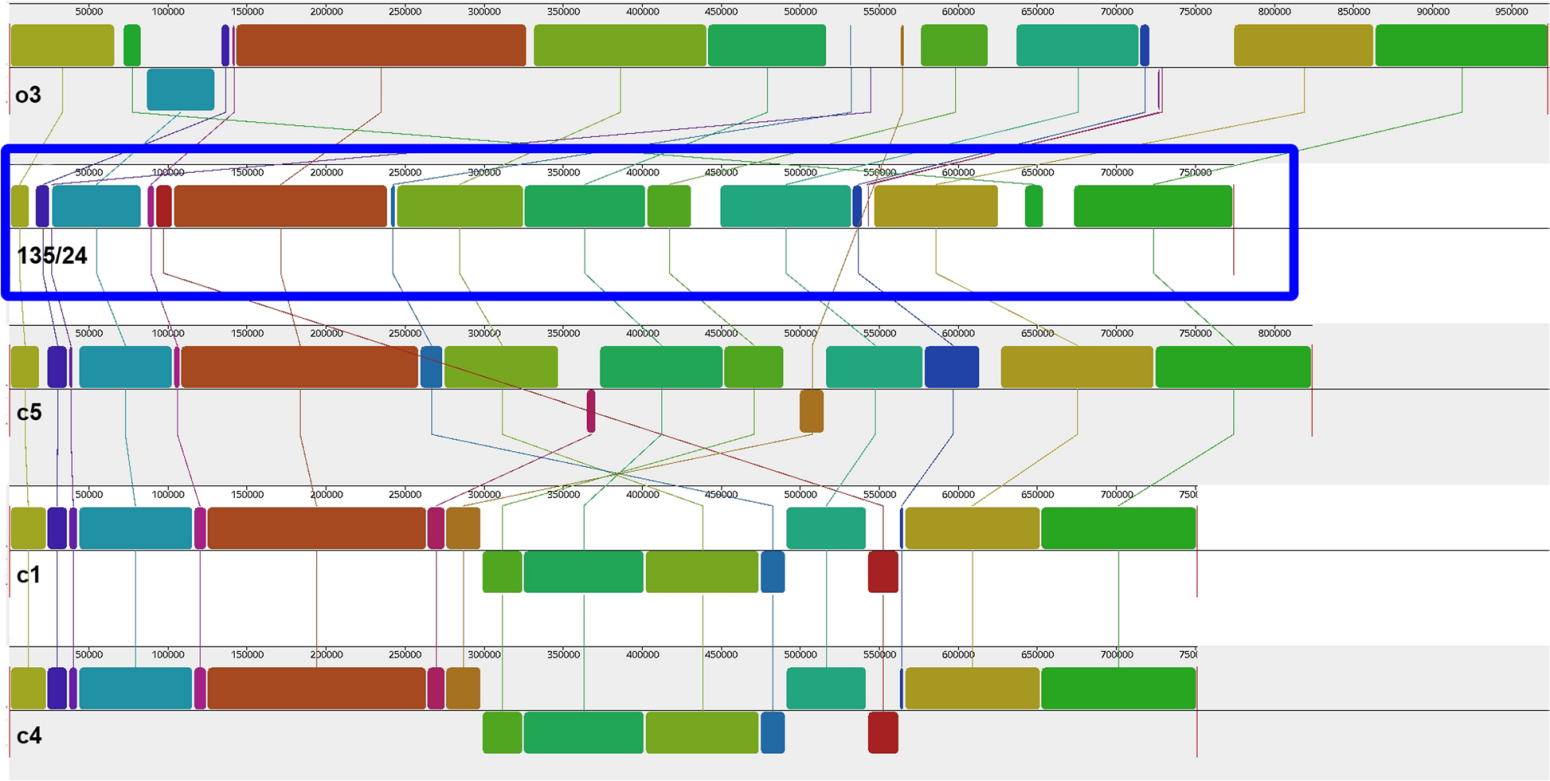
Whole-genome alignment of '*Ca*. P. solani' 135/24 (marked by the blue rectangle) with publicly available '*Ca*. P. solani' 16SrXII-A genomes (strains c5, c1, c4, and o3) obtained using Mauve. Colored regions represent Locally Collinear Blocks (LCBs), with lines connecting homologous regions across genomes, highlighting conserved chromosomal structures among closely related strains

The analyzed '*Ca*. P. solani' strains were grouped based on chromosomal collinearity. Specifically, '*Ca*. P. solani' 135/24 showed the highest collinearity with strain c5, as evidenced by blocks of conserved sequence synteny and minimal disruptions in alignment. Its collinearity with o3, c1, and c4 was less pronounced, with noticeable gaps and rearrangements. Strains c1 and c4 were highly colinear with each other (Fig. 3).

### Genome functional annotation of '*Ca*. P. solani' 135/24

**Fig. 4:**
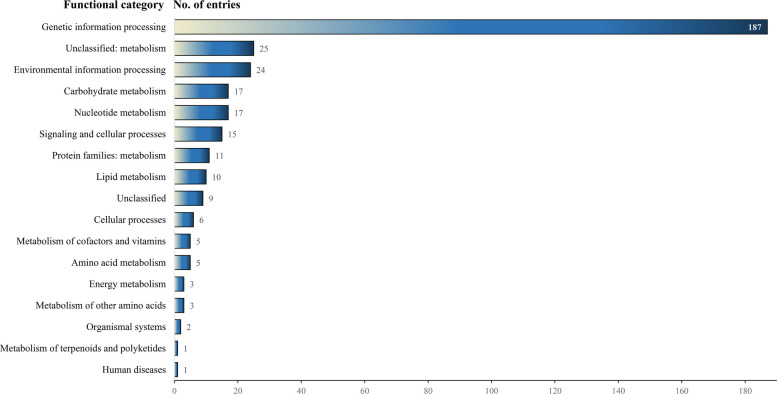
Annotated coding regions predicted in '*Ca*. P. solani' 135/24 genome sorted into functional categories

Functional annotation of the assembled genome of '*Ca*. P. solani' 135/24, performed with BlastKOALA classified coding sequences (CDSs) as KEGG Orthology (KO) entries belonging to various functional categories as listed in Fig. 4. Distribution of coding regions into functional categories showed that 58% of assigned coding regions appear associated with genetic information processing, while metabolic pathways occupy 26% of assigned coding regions.

**Fig. 5:**
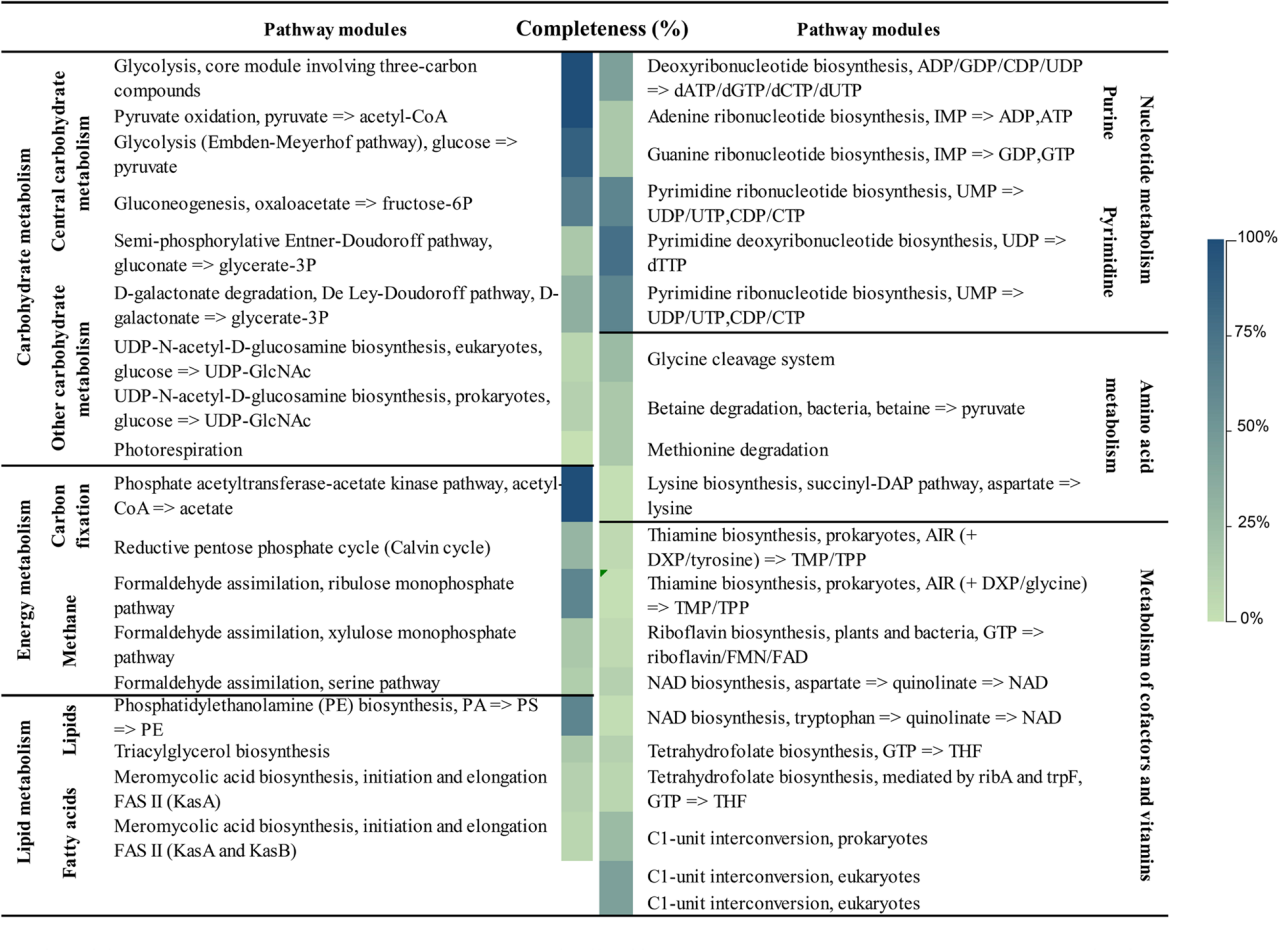
Metabolic pathway modules predicted in '*Ca*. P. solani' 135/24. Completeness (%) of each pathway is indicated by the blue color gradient. Pathways are grouped into major functional categories, including carbohydrate and energy metabolism, lipid and fatty acid metabolism, nucleotide biosynthesis, amino acid metabolism, and cofactor/vitamin metabolism

The analysis of the genome of '*Ca*. P. solani' 135/24 showed genomic features characteristic of phytoplasma of the Stolbur group (16SrXII group). In the assembled genome, we detected three complete modules (Fig. 5) related to central carbohydrate and energy metabolism, with genes associated with key metabolic pathways, as previously shown for phytoplasmas [41]. The core glycolysis pathway involving three-carbon compounds was complete and its associated genes were grouped in three operons (*pfkA-pgi*, *tpiA-fbA-pgk-gap*, and *pyk- gpmI-eno*), indicating the ability of '*Ca*. P. solani' 135/24 to convert glucose or related carbohydrates into pyruvate. Additionally, the module for pyruvate oxidation leading to the production of acetyl-CoA – which links glycolysis, the tricarboxylic acid cycle or other metabolic processes dependent on acetyl-CoA – was also annotated as complete. Another complete pathway was the phosphate acetyltransferase-acetate kinase pathway, resulting in the production of acetyl-CoA and acetate. Although phytoplasmas are wall-less bacteria, the '*Ca*. P. solani' 135/24 genome encodes incomplete pathway for fatty acid synthesis analogous to β-ketoacyl synthases *kasA* and *kasB* for biosynthesis of meromycolic acid, a precursor of cell-wall associated mycolic acid in *Mycobacterium tuberculosis* [69]. Regarding the metabolism of cofactors and vitamin pathways, genes for thiamine, NAD, THF, and riboflavin biosynthesis were also detected. Another gene typical for 16SrXII stolbur phytoplasma was found in the genome '*Ca*. P. solani' 135/24 *ribF*, which is involved in vitamin B2 synthesis, and has the highest similarity to '*Ca*. P. solani' 284/09 genome (FO393427.1).

Analyses of the genome of '*Ca*. P. solani' 135/24 revealed an RNA gene organisation typical for '*Ca*. P. solani' [15, 73]. Overall, 29 of 32 tRNAs are organized into six operons, while the remaining three are scattered individually throughout the genome. Among the six operons, four are tRNA operons, and two are mixed tRNA-rRNA operons containing 5S, 16S, and 23S rRNA genes in which the intergenic spacer region (ISR) between 16 and 23S rRNAs contains tRNA-Ile. The genome also contains a 423 bp-long tmRNA gene.

**Fig. 6:**
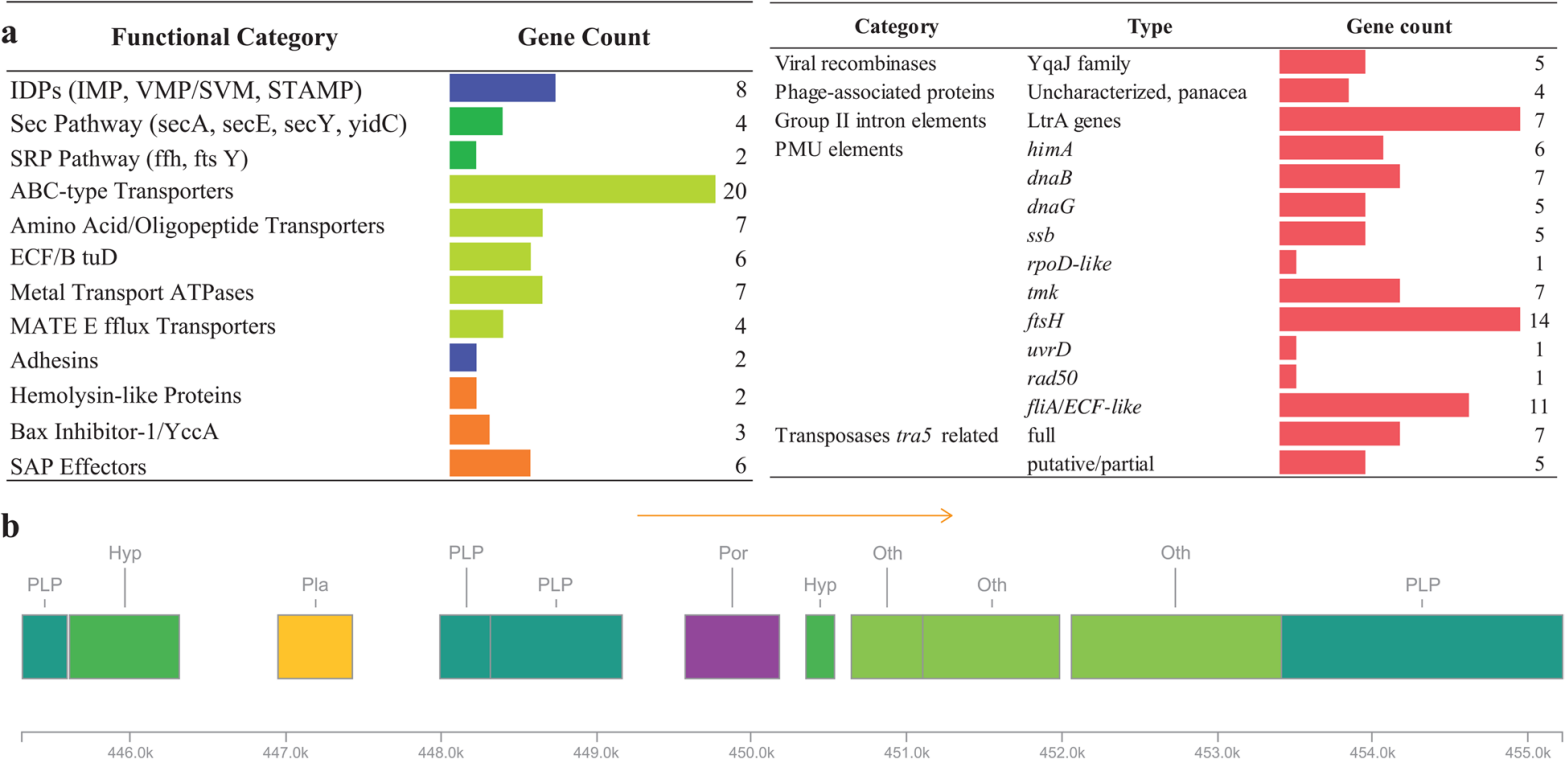
**a** Left panel – key genes in the '*Ca*. P. solani' 135/24 genome related to host–pathogen interactions. Blue bars – host–pathogen interaction proteins; dark green bars – secretion pathways; light green bars – transporters; orange bars – virulence factors. Right panel – PMU genes in the '*Ca*. P. solani' 135/24 genome. **b** Schematic representation showing the genomic organization in a prophage region identified by PHASTER. Predicted gene functions are color-coded and labeled as follows: PLP – phage-like protein; Hyp – hypothetical protein; Pla – plate protein; Por – portal protein; Oth – other

Features related to host–pathogen interactions and pathogenicity are shown in Fig. 6. Genes for immunodominant membrane proteins (IDPs) – proteins with domains exposed on the outer membrane surface, involved in host–pathogen interactions and typically present in '*Ca*. P. solani' [15], have been detected in the '*Ca*. P. solani' 135/24 genome: one immunodominant membrane protein IMP, six variable membrane proteins (VMP)/sequence-variable mosaic proteins (SVM), and one stolbur phytoplasma antigenic membrane protein gene (stamp).

The genome of the '*Ca*. P. solani' 135/24 contains a full set of Sec translocation system pathway genes (*secA*, *secE*, *secY*, *yidC*) and partial SRP pathway (*ffh* and *ftsY*) which is consistent with very similar genomes of strains c1, c4, c5, and o3 [15]. Additionally, 35 annotated genes for ATP-binding proteins related to uptake and transport systems were found: 20 associated with the ABC-type transport system (ATP-binding cassette domain-containing protein), seven associated with the amino-acid and oligopeptide transport system, six belonged to the two operons of energy-coupling factor transporter genes (ECFs) *ecfA1-ecfA2-ecfT*, and the remaining six which encoded vitamin B12 import ATP-binding proteins BtuD. Seven genes were coding for ATP-ases for Ca, Mg, Mn, and Zn transport, and four genes encoded multidrug and toxic compound extrusion family efflux transporters (MATE). Additional host–pathogen interaction-related features include the presence of genes for two adhesin-like proteins (one similar to cytadherence high molecular weight protein 2 and one similar to phytoplasma adhesin P38), two predicted channel-forming proteins related to hemolysin and hemolysin III, and three genes for virulence factor bax inhibitor-1/YccA family membrane protein – one flanked, as previously shown, by *tuf* and *rsmG* [74], and two co-located with the potential mobile unit (PMU) associated gene (*tra5* transposase) and virulence factor SAP39-like protein. In fact, the genome harbors six genes for effectors of the secreted aster yellow witches’ broom phytoplasma proteins (SAPs), of which three are SAP39-like, and three are similar to SAP50, SAP53, and SAP61.

The genome of '*Ca*. P. solani' 135/24 contains genes associated with PMU or PMU-like regions such as *tra5*, *dnaB*, *dnaG*, *fliA*, *ssb*, *rpoD*, *dam, uvrD, hflB/ftsH*, and *tmk* [15, 54, 74]. Some of these genes are present in multiple copies, and many are associated with transposable elements and prophages (Fig. 6). Accordingly, genes encoding five viral recombinases from the YqaJ family and four uncharacterized phage-associated proteins with the panacea domain were found. Throughout the genome, seven *ltrA* genes encoding group II intron reverse transcriptases/maturases were scattered. Thirteen transposases found in the genome, including five partial, were all related to transposases detected in the c1 strain and labelled as *tra5*. Apart from *tra5* transposases, '*Ca*. P. solani' 135/24 genome contained all the other genes considered as core PMU [3] (*dnaB*, *dnaG*, *tmk*, *ftsH*, *himA*, *ssb*, *rpoD-like*) and some additional PMU elements (*rad50*, *fliA/ECF-like*, *uvrD*). Most of these PMU elements were grouped, though without any discernible pattern. In total, over 20 such groups were identified as containing two to seven PMU elements often combined with virulence factors such as SAP effectors.

One incomplete prophage region, likely a relic, was identified within the '*Ca*. P. solani' 135/24 chromosome using PHASTER analysis (Fig. 2). This 9.9 kb region (positions 445,302–455,226) contains 11 protein-coding genes, including seven phage-related genes – genes for a histone-like protein, baseplate wedge, two methyltransferases, portal protein, hypothetical protein, and DNA adenine methyltransferase – consistent with a prophage-derived element, which supports its prophage-derived nature (Fig. 6).

### '*Ca*. A. phytopathogenicus' 135/24

The presence of '*Ca*. A. phytopathogenicus' was confirmed in three of five analyzed *R. artemisiae* specimens (Table 1). The number of reads assigned to ‘*Candidatus* Arsenophonus’ ranged from 2.44% (16,545 of 678,825) to 3.35% (199 of 96,850) across the samples. Seven operons containing 16S rRNA gene sequence were retrieved in '*Ca*. A. phytopathogenicus' 135/24 and showed inter-operon heterogeneity. Three distinct intragenomic 16S rRNA variants were present, occurring in three, three and one operon copies. Each variant differs from the published '*Ca*. A. phytopathogenicus' sequence (AY057392.1) associated with SBR in France by one, two and three single‑nucleotide polymorphisms (SNP), respectively. All three SNPs lie outside the fragment amplified by the Fra4/5 primer pair (positions 12, 33 and 307 of the 16S rRNA gene).

### Genome assembly and functional annotation of '*Ca*. A. phytopathogenicus' 135/24

De novo assembly of '*Ca*. A. phytopathogenicus' 135/24 yielded an 18-contig draft genome, and two putative plasmids identified as circular contigs. The draft genome of 3,157,113 bp had an average G + C content of 38.11% (largest contig, 1,961,348 bp; N50 = 1,961,348 bp, average sequencing depth 21 ×) (Table 2). Among 366 single-copy orthologue genes conserved among gammaproteobacteria (BUSCO database), 339 are present in the obtained draft genome, suggesting 92.6% complete BUSCOs. CheckM2 resulted in an even higher level of genome completeness of 100%. The assembled draft chromosome of '*Ca*. A. phytopathogenicus' 135/24 encodes 4376 coding sequences, 21 rRNA genes (seven of each 16S rRNA, 23S rRNA and 5S rRNA, organized into seven rRNA operons), 61 tRNA genes, and one tmRNA gene (Table 2).

**Fig. 7:**
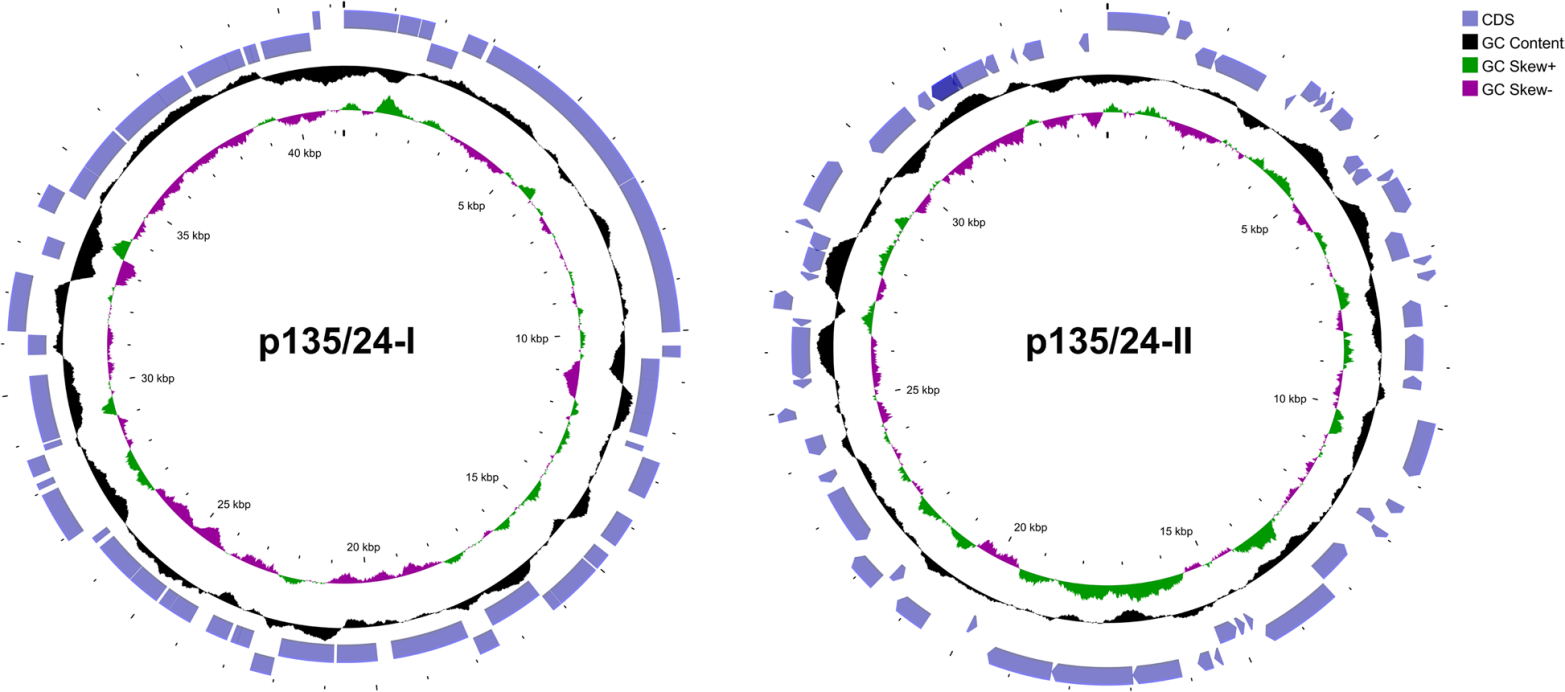
Map of the circular extrachromosomal/putative plasmid elements in the genome of '*Ca*. A. phytopathogenicus' 135/24 from *R. artemisiae* 135/24

Two putative plasmids, p135/24-I and p135/24-II, with sequence lengths of 41,261 bp and 34,756 bp, respectively (Fig. 7), were identified according to the circular nature of their assembly and higher sequencing depth (70 × and 41 × coverage, respectively) compared to the chromosomal average. Putative plasmid contigs were associated with '*Ca.* A. phytopathogenicus' based on binning consistency and taxonomic annotation. Their plasmid identity was further supported by the presence of plasmid-related genes, including the Rep3 replication initiation protein (Fig. 7). Position one (*ori*) for each of the two plasmids was set to the first nucleotide of gene *rep3*.

Rings from the outside to inside represent: (1, 2) predicted protein-coding genes on the forward and reverse strands, respectively; (3) GC content, (4) GC skew, with regions above the average shown in green and below average in purple; (5) plasmid coordinates (kb).

When compared to publicly available genomes of '*Ca*. A. phytopathogenicus' to establish average nucleotide identity, the '*Ca*. A. phytopathogenicus' genome originating from *R. artemisiae* 135/24 showed close relatedness (above 99.5%) to all three publicly available strains (Table 4).

**Table 4:** Comparative genomic features of '*Ca*. A. phytopathogenicus' 135/24 and publicly available '*Ca*. A. phytopathogenicus' genomes

Strain	**135/24**	Ap-CH	Ap-Fr	PENLEP
Acc. Number	JBUIOB000000000	GCA_047291415	GCA_047291325	N/A^a^
Contigs	18	33	67	1
ANI with 135/24 (%)	100	99.81	99.66	99.86
Length (bp)	3110796	2875309	2583717	3075959
GC (%)	37.96	37.68	37.08	37.6
CDS	3,968	3213	2710	3802
CheckM2 completeness (%)	100	99.46	96.76	N/A
rRNAs (5S, 16S, 23S)	21(7,7,7)	21	7	N/A
Number of tRNAs	61	61	47	N/A
Plasmids (size in bp)	p135/24-I (41261) p135/24-II (34756)			Plasmid 1 (71678) Plasmid 2 (43054) Plasmid 3 (40921) Plasmid 4 (39723)

^a^N/A-not available, genomic features of PENLEP have been previously published [78]

**Fig. 8:**
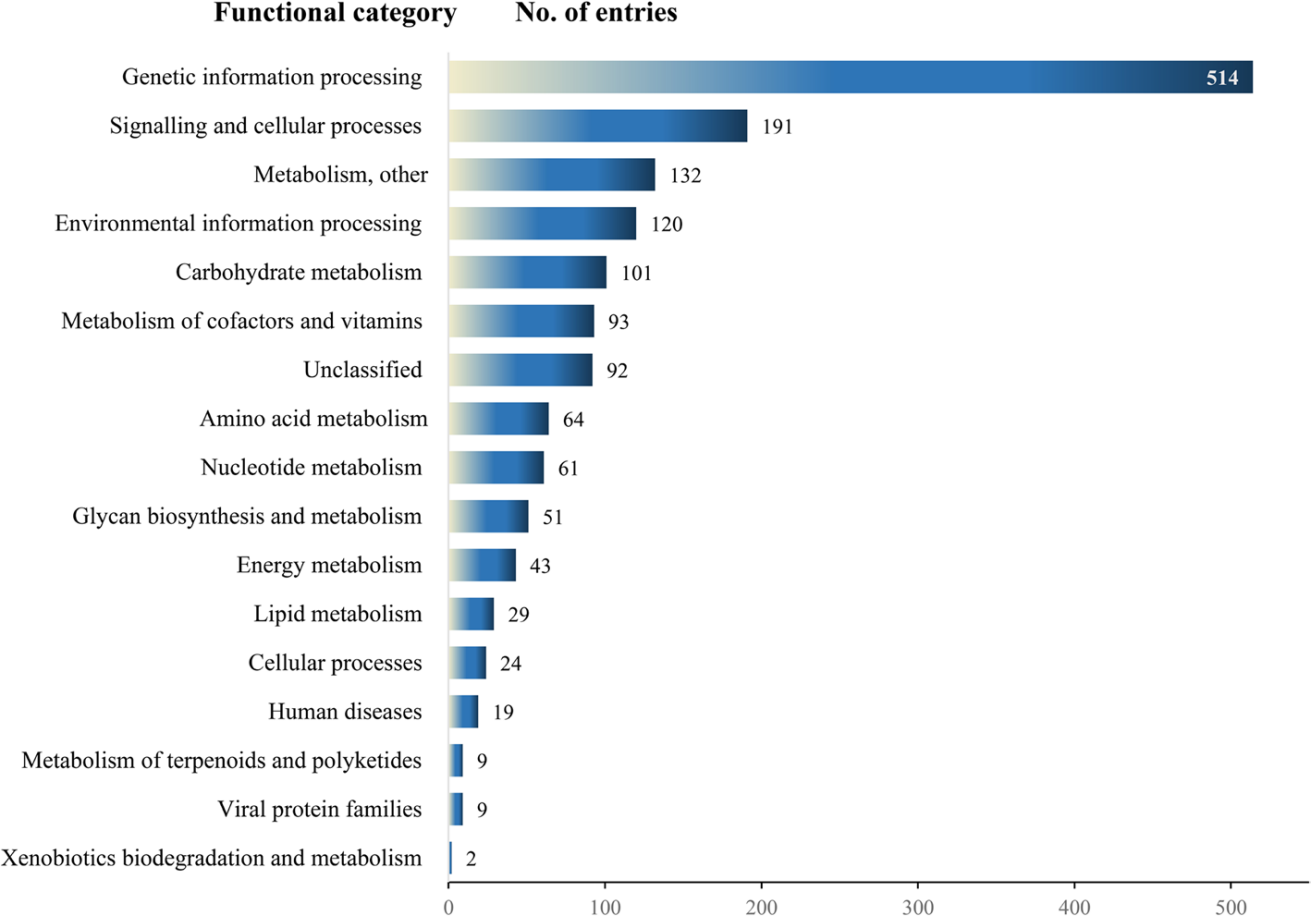
Annotated coding regions predicted in '*Ca*. A. phytopathogenicus' 135/24 genome sorted into functional categories

The functional annotation classified 1,559 KO entries into 17 categories, among which a category labelled as unclassified contains 92 KO entries (Fig. 8). Most of these assigned coding sequences were annotated as components of metabolic pathways (37.5%) or genetic information processing (33%), and attributed to either specific proteins, protein families, or members of the unclassified category. Categories of metabolic pathways are shown in Fig. 8. Additionally, the following was found within the '*Ca*. A. phytopathogenicus' 135/24 genome: four RNA polymerase genes, 80 genes involved in translation processes (56 ribosome biogenesis genes and 24 genes involved in aminoacyl-tRNA biosynthesis), 46 genes involved in protein folding, sorting, and degradation, and 81 gene involved in DNA replication and repair. Signalling and cellular processes represent up to 14% of KO entries and consist of 82 genes involved in membrane transport, 70 genes involved in signal transduction, and 96 genes involved in cellular processes, of which 13 genes are involved in cell motility. Among 61 detected tRNA genes, three associated with the TTA codon are non-functional. Additionally, a single gene found for a suppressor tRNA molecule is also TTA-associated. Furthermore, tRNA genes were single or organized in small operons consisting solely of tRNA genes or in mixed operons with 5S, 16S, and 23S rRNA genes. KEGG pathway mapping assigned 19 '*Ca*. A. phytopathogenicus' 135/24 genes to various “bacterial infectious disease” pathways, which reflects the presence of homologous secretion of adhesion and invasion proteins that are conserved among Gram-negative intracellular bacteria.

**Fig. 9:**
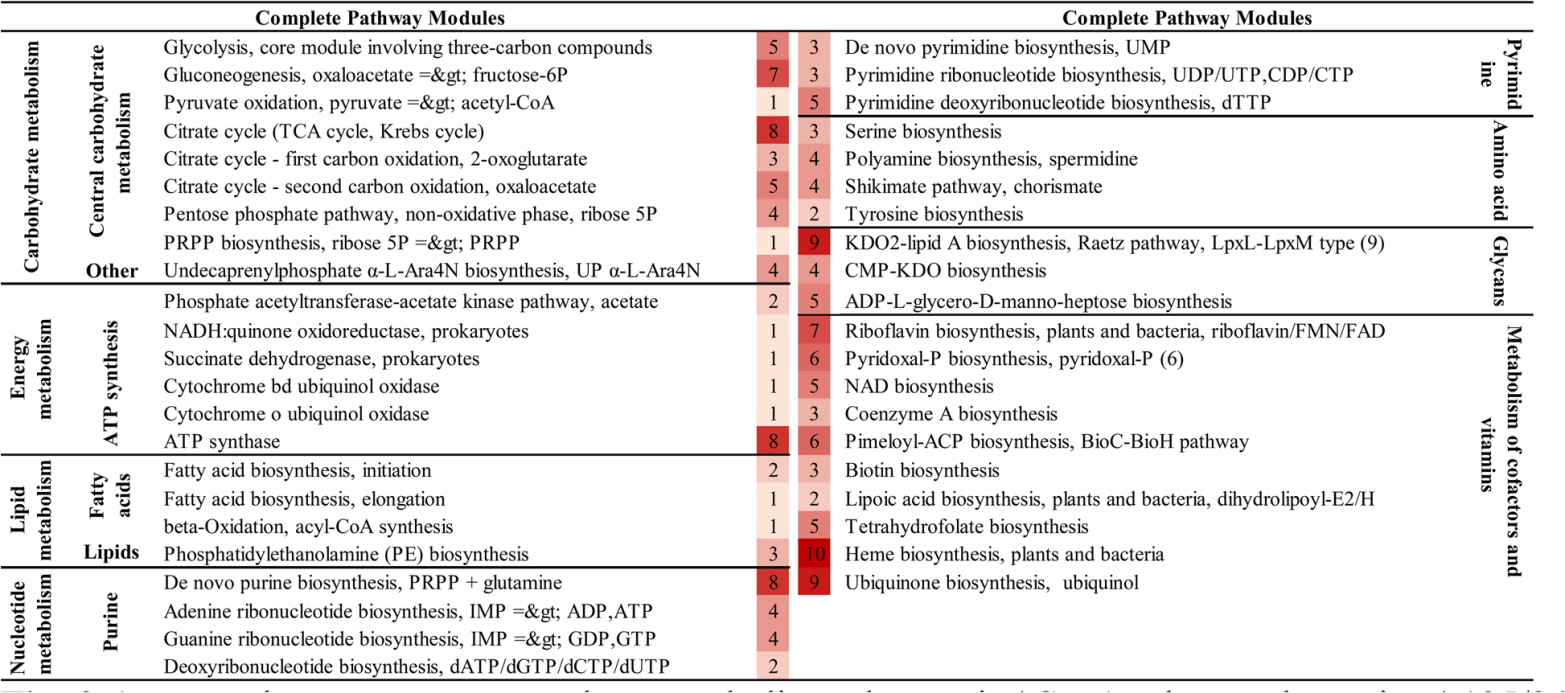
Annotated genes across complete metabolic pathways in '*Ca*. A. phytopathogenicus' 135/24. Pathways are grouped by functional category including central carbon metabolism, energy generation, lipid and fatty acid metabolism, nucleotide metabolism and the pentose phosphate/PRPP biosynthesis pathway, amino acid and polyamine metabolism, glycan and polysaccharide biosynthesis, and cofactor and vitamin biosynthesis, with color intensity reflecting the gene counts per pathway

Compared to the genome of '*Ca*. P. solani' 135/24 which contains only three complete metabolic pathways, the genome of '*Ca*. A. phytopathogenicus' 135/24 supports a much more robust metabolism, with numerous predicted complete pathways. The pathways detected and denoted as complete within the '*Ca*. A. phytopathogenicus' 135/24 genome are shown in Fig. 9.

The presence of complete core pathways, such as those for glycolysis, gluconeogenesis, the TCA cycle, and the pentose phosphate pathway, implies a retained capacity for basic carbohydrate metabolism in '*Ca*. A. phytopathogenicus' 135/24. Other abundant functional groups represented various pathways connected to cofactor and vitamin biosynthesis. The presence of NADH:quinone oxidoreductase and terminal oxidases suggests electron transport capacity within '*Ca*. A. phytopathogenicus' 135/24. However, its genome lacks complete pathways related to the synthesis of amino acids such as asparagine, glutamine, or branched-chain amino acids (leucine, isoleucine, valine).

Scattered throughout the genome of '*Ca*. A. phytopathogenicus' 135/24 were genomic islands (GIs) that contain mobile genetic elements, phage-related sequences, and pathogenicity/virulence factors, grouped into small pathogenicity islands. GIs were visualized when the genome of '*Ca*. A. phytopathogenicus' 135/24 was compared to the closest publicly available reference genome of *A. nasoniae* FIN strain (Supplementary Fig. 1). In total, 28 GIs were predicted by at least one GI prediction method either by SIGI-HMM or IslandPath-DIMOB. Detected genes involved in virulence and pathogenicity included a complete set of genes of the type III secretion system (T3SS) apparatus, comprising inner and outer membrane proteins, homologues of the cell invasion protein SipD, flagellar proteins, membrane channel proteins, ATPases, and effector proteins. Other genes involved in virulence and pathogenicity were *iagA* (coding for a transcriptional regulator of invasion), genes for a putative insecticidal toxin complex, a putative large exoprotein involved in heme utilization or adhesion of the ShlA/HecA/FhaA family, a virulence sensor protein BvgS precursor, a homologue of murine toxin with a phospholipase D-like domain, and multiple genes for RTX toxin RtxA with corresponding transporters.

Transposases detected in the '*Ca*. A. phytopathogenicus' 135/24 genome belong to two families, IS200/IS605 transposases (ISEc46, ISEc42, ISSen6) and ISNCY transposases (ISSen7, ISPlu15, ISRor2), with duplicates for ISEc46 and ISPlu15 suggesting either multiple genomic copies or annotation repeats. Due to fragmentation in assembly resulting from repetitive regions, it is difficult to assess the correct number of genetic determinants, as the annotation contained repeats of the same gene, interrupted with short “unknown proteins”.

Approximately 29% of the annotated genes in the '*Ca*. A. phytopathogenicus' 135/24 genome were of phage origin, including those encoding viral structural proteins (tail, head, and portal), integrases/recombinases, lytic enzymes (lysozyme/holine), and proteins involved in DNA replication, stabilization, and transfer, as well as other phage-related functions organized within GIs. PHASTER estimated the presence of 26 phage-derived regions distributed across 14 contigs, comprising seven intact, 16 incomplete (probable relics), and three questionable regions. The complexity of phage-sequence-containing GIs varied from small, scar-like remnants containing only partial integrase genes and a few phage sequences, to putative prophage regions harboring numerous genes involved in replication, capsid assembly, and packaging, as well as full-length integrase genes. Several of these integrase genes were located adjacent to tRNA loci, consistent with well-known integration hotspots [84]. Three such regions contained the *polA* gene – one fragmented and two sharing 91% sequence identity with the *Hamiltonella* phage APSE-1 sequence [9, 48]. Chromosomal phage-derived segments ranged in size from 8.5 to 118.1 kb, while two phage-derived regions, an 11.8 kb incomplete region and a 12.5 kb questionable region, predicted to be located within p135/24-I together covered approximately 60% of the plasmid.

Eight of 20 '*Ca*. A. phytopathogenicus' 135/24 assembly contigs, totalling 169,746 bp (representing 5.46% of the genome), could not be aligned to the *A. nasoniae* FIN genome. These unaligned contigs included four GIs, among which was the largest GI identified in the assembly. This region exhibited a complex organization and contained multiple mobilome-associated elements, including phage sequences, transposases, and plasmid-related sequences showing similarity to plasmids paPv2, paPv3, and paPv5 of *Arsenophonus nasoniae* strain aPv. In addition, this region carried multidrug-resistance genes and genes involved in plasmid maintenance, partitioning, and conjugative transfer. Within these unaligned regions, two putative circular plasmids (p135/24-I and p135/24-II) were located (Fig. 7). A putative integrated plasmid 35,912 bp in length was also identified based on the presence of numerous replication, partitioning, and conjugation-associated genes interspersed with phage and transposase sequences. This genomic region contained *incF*-type conjugative transfer genes, the ParA-like protein gene, several origin-specific replication binding factors, and the TrfA-related protein gene. The presence of phage-plasmid mosaic organization, transposase genes (including InsQ-like elements) and mobile element proteins supports the interpretation that this chromosomal region was formed as a result of a plasmid integration event.

## Discussion

In this study we employed PCR-free metagenomic long-read sequencing (ONT MinIon) to obtain a relatively unbiased insight into the microbiome of five *R. artemisiae* specimens. Use of ONT long-read sequencing approaches for highly accurate, long-read-only assembly has already been demonstrated for bacterial metagenome assembled genomes [46, 64, 86]. Although this approach avoids biases inherent to selective amplification techniques, which target specific gene regions (e.g., 16S rRNA), and dependency on primer specificity [44, 61], it nevertheless introduces selection bias through reliance on a custom database for taxonomic profiling. In addition, variability in sequencing depth, caused by singleplex versus multiplex runs and differences between the first and second flow-cell runs, produced uneven data output among samples (e.g., 2.5 Gb for specimen 135/24 versus 8.8 Mb for 69/24). Consequently, this study does not provide quantitative data or a comprehensive overview of the entire *R. artemisiae* microbiome, but rather a qualitative assessment of the most important and abundant associated microbes.

Phloem-sap-feeding vectors, such as leafhoppers and planthoppers, often harbor a diverse microbial consortium, including primary symbionts for essential nutrient provisioning and secondary associates (secondary endosymbiont/plant pathogen like '*Ca*. A. phytopathogenicus' or plant pathogens like '*Ca*. P. solani'). While there are published data about microbial communities of two other plant pathogen-vector cixiids [10, 28, 29, 78], this is the first study of the microbial community of *R. artemisiae*, the dominant vector of '*Ca*. P. solani' on sugar beet in the Pannonian Plain. Our findings revealed that up to six bacterial taxa spanning five classes can be present in a single *R. artemisiae* specimen—Mollicutes ('*Ca*. P. solani'), α-proteobacterium (*Wolbachia*), β-proteobacterium ('*Ca.* Vidania'), γ-proteobacteria ('*Ca*. A. phytopathogenicus' and '*Ca.* Purcelliella') and Flavobacterium ('*Ca.* Karelsulcia').

Previous studies of cixiid vectors have identified four bacteria in *P. leporinus*, up to five in *H. obsoletus* [10, 28, 29], and, most recently [78], a diverse multi-partner community of seven bacterial taxa, including rickettsia, in *P. leporinus*. Identification of four endosymbionts (*Wolbachia*, *Vidania*, *Purcelliella*, *Karelsulcia*) in all specimens of *R. artemisiae* evaluated in this study aligns with previous findings [10, 28, 29, 78], as these endosymbionts have been co-detected alongside '*Ca*. P. solani' in *H. obsoletus*, and '*Ca*. P. solani' and '*Ca*. A. phytopathogenicus' in *P. leporinus*. These same four endosymbionts have also been observed in other cixiid species, including *H. luteipes*, *P. rorida* and *C. nervosus* [49]. The ubiquity of primary endosymbionts in phloem-sap feeders is expected as they are essential for the insect host. However, the presence of the secondary endosymbiont *Wolbachia* in all evaluated *R. artemisiae* specimens also appears common, as its presence was reported for 488 specimens of another '*Ca.* P. solani' cixid vector, *H. obsoletus* [28]. The potential for coexistence of six prokaryotes within a single insect host highlights the complexity of microbial relationships, ranging from mutualistic complementation to competitive exclusion within the host’s bacteriome [28]. These interactions may modulate nutritional fitness, immune responses, and reproductive success of the insect, ultimately enhancing or constraining the transmission efficiency of plant pathogens. Such findings emphasize that horizontal symbiont acquisition and vertical transmission shape evolutionary trajectories, potentially driving pathogen spread in agroecosystems. Because insect microbiome and pathogen strains can vary by geography, season, and host plant, the small sample size (*n* = 5), single-season sampling, and single-site collection preclude drawing robust ecological conclusions or making reliable assessments of prevalence, co-infection patterns, and population-level variability.

The results of our study confirm a strong phylogenetic congruence between the three primary endosymbionts and their insect hosts, consistent with earlier findings [28, 53, 77]. This congruence supports the hypothesis of ancient symbiont acquisition by a shared ancestor of these insects, followed by subsequent coevolution driven by vertical transmission [53]. In contrast, plant pathogens and the secondary insect endosymbiont, *Wolbachia*, appear to follow independent evolutionary trajectories. While these pathways of plant pathogens are naturally influenced by dual-host dynamics and vector-mediated transmission across multiple plant species [76], the absence of phylogenetic congruency of *Wolbachia* with its insect hosts indicates that it has been acquired by hosts recently, and/or may be transferred among insect species horizontally. Plant-mediated horizontal transmission of *Wolbachia* between phloem-sap-feeding insects has already been documented [45].

While *H. obsoletus* is an established vector of '*Ca*. P. solani' and *P. leporinus* transmits both '*Ca*. P. solani' and '*Ca*. A. phytopathogenicus'*, R. artemisiae* was previously known to transmit only '*Ca*. P. solani' and has recently been shown in Austria to also transmit '*Ca*. A. phytopathogenicus' [38, 40]. The current study confirms that *R. artemisiae* harbors '*Ca.* A. phytopathogenicus' alongside '*Ca.* P. solani' in the Pannonian Plain (Serbia), but detection of pathogen in insect bodies alone does not demonstrate transmission to host plants in this region. Thus, although our results raise important questions regarding the potential contribution of *R. artemisiae* to '*Ca*. A. phytopathogenicus' circulation in areas where *P. leporinus* is absent, definitive assessment of transmission capacity of *R. artemisiae* and its epidemiological implications in *P. leporinus*-free areas requires further research, including controlled transmission assays.

In addition to characterizing the endomicrobiota of *R. artemisiae*, this study provides new genomic resources, including a complete genome of '*Ca*. P. solani' (16SrXII-A) and a draft genome of '*Ca*. A. phytopathogenicus'. The chromosome of '*Ca*. P. solani' 135/24 has a circular organisation, common for '*Ca*. P. solani' and most other phytoplasma genomes, with the exception of those belonging to 16SrX [42]. The size of the obtained chromosome, as well as the number of CDSs, G + C content, coding and gene densities are within the range of other '*Ca*. P. solani' strains (Table 3). The number and organization of RNA genes obtained for '*Ca*. P. solani' 135/24 is consistent among all '*Ca*. P. solani' genomes [15, 73]. The BUSCO and CheckM2 analyses indicate high-quality assembly, as the obtained completeness levels are consistent with publicly available '*Ca*. P. solani' genomes [15, 73]. While the ANI analyses of the complete '*Ca*. P. solani' genomes clustered together strains belonging to the 16SrXII-A subgroup, they provided a distinct placement for the 16SrXII-P strain. Whole-genome alignment of '*Ca*. P. solani' 135/24 genome with publicly available '*Ca*. P. solani' 16SrXII-A genomes, based on chromosomal collinearity, highlighted conserved chromosomal structures among closely related strains. Specifically, '*Ca*. P. solani' 135/24 showed the highest collinearity with strain c5, as evidenced by blocks of conserved sequence synteny and minimal disruptions in alignment. Its collinearity with o3, c1, and c4 was less pronounced, with noticeable gaps and rearrangements, suggesting greater genomic divergence. Collinearity of '*Ca*. P. solani' 135/24 with strains c1 and c4 was identical (Fig. 3), as the latter two are considered to be the same strain [15]. The visual representation in Fig. 3 underscores not only genetic relationships, but also structural variations within the '*Ca*. P. solani' 16SrXII-A ribosomal group.

The functional reconstruction of '*Ca*. P. solani' 135/24 genome showed genomic features characteristic of phytoplasma of the Stolbur group (16SrXII group). Blast KOALA classified 325 CDSs as KEGG Orthology entries, which is similar to the outcome obtained for the closest related strains – c1 (357), c4 (358), c5 (366), and o3 (423) [15]. In the assembled genome, three complete modules (Fig. 5) related to central carbohydrate and energy metabolism were predicted, including genes associated with key metabolic pathways, as previously shown for phytoplasmas [41]. Prediction of pathway modules for glycolysis, 3-carbon metabolism, and pyruvate oxidation through acetyl-CoA to acetate suggests retention of a streamlined but functional central carbon metabolism that can process sugars imported from host phloem-sap to acetyl-CoA, while the acetate kinase/phosphate acetyltransferase pathway allows regeneration of ATP. This finding is consistent with the established idea that phytoplasmas lack oxidative phosphorylation and depend mainly on glycolysis/fermentation for energy [59, 75, 81]. Their ability to produce ATP is further supported by the presence of 88.89% of genes of the glycolysis (Embden-Meyerhof) pathway, enabling energy generation via substrate-level phosphorylation. As in most phytoplasmas, these genes are clustered and are located in three operons in '*Ca*. P. solani' 135/24. The near-completeness of this pathway suggests that it is functional; however, the hexokinase gene seems absent in '*Ca*. P. solani' 135/24, thus the glycolysis pathway may rely on host-derived imported glucose-6-phosphate. This prediction is consistent with findings for many obligate intracellular bacteria (including phytoplasmas), which often lack hexokinase and rely on host metabolites to fuel glycolysis [41]. Pyrimidine deoxyribonucleotide biosynthesis (UDP to dTTP) is represented with 80% genes of the pathway that is key for DNA replication and repair. This “almost complete” pathway suggests that '*Ca*. P. solani' 135/24 still retains some of its nucleotide metabolism, though the severe incompleteness of ATP and GTP synthesis pathways (25%) and moderate incompleteness of pyrimidine (CTP, UTP, dTTP) synthesis pathways (67%, 67%, and 80%, respectively) imply that '*Ca*. P. solani' 135/24, as many other phytoplasmas, relies on scavenging bases/nucleosides from its host [58, 70, 85]. Analysis of genes for membrane-associated proteins indicates that '*Ca*. P. solani' 135/24 is a pathogen well-equipped for intracellular survival, immune evasion, and manipulation of host physiology. The presence of IMP, VMPs/SVMs, and Stamp genes, with a strong role in immune evasion and antigenic variation, suggests an expanded toolkit for evading host defences compared to related strains. Though the genome of '*Ca*. P. solani' 135/24 encodes six SAP effectors, including SAP39-like, SAP50, SAP53, and SAP61 – more than strains c1, c5, or o3 [15] – the true complement may be larger, as SAP family members can have very low pairwise amino-acid identity (as low as 37%), which possibly enables highly divergent homologs to escape detection by similarity-based annotation. As SAPs are known to reprogram plant development and immunity – e.g., SAP54 destabilizes MADS-box transcription factors, causing phyllody [47] – the diversity and abundance of SAPs may affect the pathogenic versatility of '*Ca*. P. solani' 135/24, allowing it to modulate plant growth and immunity in multiple ways. Mobile genetic elements contribute to effector diversity in phytoplasmas by transferring them within PMUs [50]. The genome of '*Ca*. P. solani' 135/24 contains 13 *tra5*-related transposases, as well as LtrA group II intron maturases. The presence of viral recombinases and phage-associated proteins further points to horizontal gene transfer and prophage activity. Such mobile element richness indicates a dynamic genome with ongoing rearrangements and gene acquisitions/losses. While the export of virulence proteins (including SAPs) and pathogenicity factors into the host cell is supported by the complete Sec pathway in '*Ca*. P. solani' 135/24, this strain, unlike c1, c5, and o3, possesses a reduced SRP pathway. However, the membrane of '*Ca*. P. solani' 135/24 supports multiple proteins for transport and nutrient acquisition, such as ABC-type transporters, oligopeptide/amino acid systems, ECFs, and vitamin B12 importers, allowing this strain to compensate for its metabolic limitations by heavy reliance on host-derived metabolites [58, 70]. Though all transporter types uncovered in '*Ca*. P. solani' 135/24 were typical for Stolbur phytoplasmas [74], their exact numbers differed from those detected in the GOE strain, as '*Ca*. P. solani' 135/24 was richer in metal transport ATP-ases (7 in '*Ca*. P. solani' 135/24, as compared to 4 in GOE). The presence of virulence factors in '*C*a. P. solani' 135/24 emphasizes its ability for stable colonization through adhesion, host manipulation, and suppression of host defences. This strain possesses entire arsenal of proteins for successful binding (adhesion-like proteins), modulation of host membrane permeability (hemolysin-like proteins), and suppression of host-programmed cell death (Bax inhibitor/YccA proteins), allowing '*Ca*. P. solani' 135/24 to maintain long-term infections [8, 55, 80].

Although long-read sequencing was employed, the '*Ca.* A. phytopathogenicus' 135/24 genome assembly remains at the scaffold level, comprising 20 contigs with a total length of 3.16 Mbp and 92.6% BUSCO completeness (Table 2), likely due to its highly repetitive nature. CheckM2 estimated the draft assembly's completeness at 100% based on conserved marker genes. However, biases from uneven marker distribution across the chromosome and under-representation of complete *Arsenophonus* genomes in databases likely inflate this estimate, warranting caution in its interpretation. Presently, all published genome sequences of '*Ca.* A. phytopathogenicus' strains are from *P. leporinus*; no sequence from any strain from *R. artemisiae* has been deposited to date. Moreover, no complete '*Ca.* A. phytopathogenicus' genome is publicly available for comparison, although a full genome is in press [78]. The best publicly available reference is the draft genome of strain AP-CH from Switzerland, with a size of 2.86 Mbp [48]. Comparative analysis of global genomic features, such as chromosome size, G + C content, and the number of coding sequences (Table 4), indicates that '*Ca.* A. phytopathogenicus' 135/24 is highly similar to strain AP-CH and the unpublished PENLEP genome [48, 78]. In this study two putative plasmids were identified in the '*Ca.* A. phytopathogenicus' 135/24 genome, compared to four reported in the PENLEP strain. Considering that '*Ca.* A. phytopathogenicus' 135/24 is represented by an incomplete draft genome, it is not excluded that more plasmids may yet be present in this strain, particularly since several plasmid-related genes were detected in other non-circular contigs, including genes encoding the TrfA-related protein, ParA-like partitioning protein, IncF plasmid conjugative transfer proteins (TraU, TraN, TraH, TraI), small multidrug resistance (SMR) family protein, RelE/StbE replicon stabilization toxin, and multiple transposases. Plasmids, together with other mobile elements such as phages, play important roles in the evolution and ecology of bacteria through facilitating the transfer of genetic material within or between genomes and are even considered as key contributors to bacterial evolution [30, 60]. In addition to at least two plasmids the genome of '*Ca.* A. phytopathogenicus' 135/24 harbors 26 phage-related regions, while the draft genome of AP-CH predicts only 18. It is not uncommon for an *Arsenophonus* species to harbor even more mobile elements as *A. nasonie* contains as many as 17 extrachromosomal genetic elements and 50 genomic regions that represent either intact or degraded phage material [24, 48]. The presence of prophage regions in plasmids is not uncommon – some *Arsenophonus* extrachromosomal elements were recently characterized as “phage-plasmids”, which harbor both phage and plasmid modules. In '*Ca.* A. phytopathogenicus' 135/24, approximately 60% of plasmid p135/24-I was annotated as prophage region; previously, no more than 50% of a plasmid has been annotated in such a manner for the genus *Arsenophonus* [60]*.* Despite the presence of numerous mobile genetic elements, suggesting that '*Ca.* A. phytopathogenicus' 135/24 is undergoing an active phase of genome rearrangement and adaptation [5], the genome of this strain retains a nearly complete repertoire of core metabolic pathways. However, several peripheral carbohydrate pathways, such as Entner-Doudoroff variants, glucuronate and galacturonate degradation, and glycogen turnover, are incomplete, indicating limited flexibility in utilizing diverse sugars [31]. Energy metabolism is robust for oxidative phosphorylation and ATP synthesis, but incomplete for alternative cycles, which may restrict utilization of unusual carbon sources [25]. Lipid and nucleotide metabolism are mostly intact, supporting membrane and nucleic acid biosynthesis, while amino acid metabolism is uneven. Core routes like serine, aromatic amino acid, and purine/pyrimidine biosynthesis are complete, but methionine, lysine, branched-chain amino acid, and proline pathways lack certain steps, potentially leading to auxotrophy [66]. Similarly, several cofactor and vitamin biosynthesis routes (e.g., thiamine, biotin, lipoic acid, NAD) are incomplete, implying possible environmental dependency or reliance on salvage pathways. *Arsenophonus* spp. vary widely in genome size, and gene loss occurs rapidly during the transition to vertical transmission, as highlighted by Siozios et al. [68]. *A. nasoniae* FIN, cultivable in vitro and the closest species with a complete genome available, possesses complete Entner-Doudoroff and glycogen turnover pathways, based on the KEGG pathway predictions, but same as '*Ca.* A. phytopathogenicus' 135/24, lacks complete pathways for methionine, lysine, and branched-chain amino acid biosynthesis, as well as for thiamine, biotin, lipoic acid, NAD biosynthesis, and glucuronate and galacturonate degradation. It should be noted that 135/24 genome is currently a draft, so some sequences may be missing, and the apparent differences in pathway completeness may therefore be overestimated. Such a combination of a dynamic mobilome and self-sufficient metabolic potential of '*Ca.* A. phytopathogenicus' 135/24 indicates a bacterium in transition, maintaining broad metabolic autonomy, while actively acquiring or reorganizing its genetic material, possibly in response to changing ecological niches and host interactions [25, 51, 83]. Absence of certain pathway modules suggests metabolic constraints that could limit bacterial survival under nutrient-limited or variable conditions, and might create dependencies on the host or environment for certain amino acids, cofactors, or secondary metabolites [68]. Conserved metabolic pathways, genomic islands, and virulence factors, including type III secretion system genes, highlight a shared evolutionary trajectory of the '*Ca.* A. phytopathogenicus' 135/24 associated with *R. artemisiae* and other cixiid vectors.

## Conclusion

This study provides the first comprehensive characterization of the microbiota of *Reptalus artemisiae*, revealing a stable consortium of primary and secondary endosymbionts coexisting with two plant pathogens, '*Ca.* P. solani' and '*Ca.* A. phytopathogenicus'. The consistent presence of multiple endosymbionts and the variable occurrence of pathogens within a single host underscore the ecological and evolutionary complexity of these associations. A deeper understanding of the molecular mechanisms and role of symbiotic bacteria, based on the rapid development of -omics techniques will provide a macro-scale perspective for studying the entire interaction network. The detailed characterization of *R. artemisiae* endosymbionts provided here offers targets for novel control strategies (e.g. *Wolbachia*-based suppression) that could reduce vector competence and limit the spread of plant pathogens. The genomic data obtained for both pathogens provide valuable resources for comparative and functional analyses, offering new perspectives on their adaptation, transmission, and epidemiology.

## Supplementary Information

Supplementary Material 1.

## Data Availability

All sequences analyzed in the present study can be accessed in the NCBI GenBank database under the bio project accession number (PRJNA1215974).
