# Supplementary material for: Microbial diversity of plant pathogens and insect endosymbionts in Reptalus artemisiae
Source: BMC Microbiol. 2026 Mar 13;26:502. doi: 10.1186/s12866-026-04915-x (PMC13202766; doi:10.1186/s12866-026-04915-x)
Supplement: Supplementary file 1 — Supplementary Material 1. [file 12866_2026_4915_MOESM1_ESM.docx]

**Supplementary material**

**Figure S1**


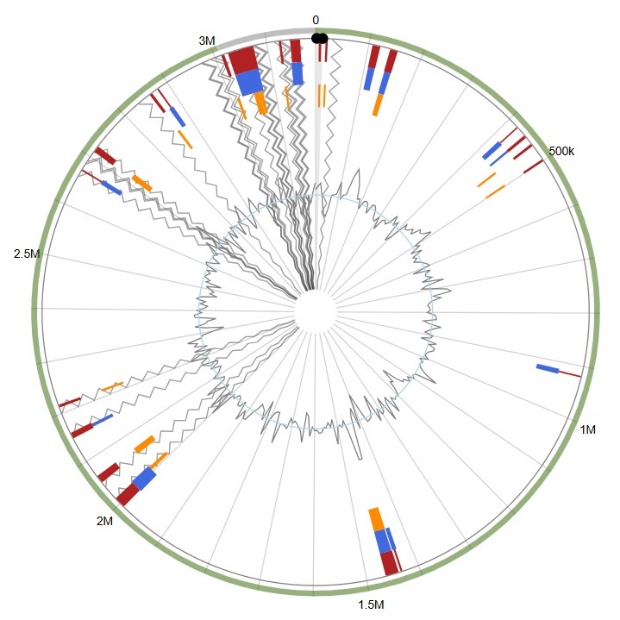


**Fig. S1** Genomic islands in the '*Ca*. A. phytopathogenicus' assembly aligned to the *A. nasoniae* FIN genome. GIs predicted by SIGI-HMM are shown in orange, those predicted by IslandPath-DIMOB in blue, and GIs predicted by one or more tools are displayed in the integrated view as red regions.
